# Public Use of the “Your COVID Recovery” Website Designed to Help Individuals Manage Their COVID-19 Recovery: Observational Study

**DOI:** 10.2196/37811

**Published:** 2023-01-20

**Authors:** Molly M Baldwin, Enya Daynes, Emma Chaplin, Amye Goddard, Phoebe H I Lloyd-Evans, George Mills, Annabel Hong, Nikki Gardiner, Sally J Singh

**Affiliations:** 1 Centre for Exercise and Rehabilitation Science NIHR Leicester Biomedical Research Centre - Respiratory University Hospitals of Leicester Leicester United Kingdom; 2 Department of Respiratory Sciences University of Leicester Leicester United Kingdom

**Keywords:** COVID-19, coronavirus, pandemic, symptom management, digital healthcare, Google Analytics, website analysis, digital health tool, user behavior, healthcare platform

## Abstract

**Background:**

At the start of the COVID-19 pandemic, unprecedented pressure was placed on health care services globally. An opportunity to alleviate this pressure was to introduce a digital health platform that provided COVID-19–related advice and helped individuals understand and manage their COVID-19 symptoms. Therefore, in July 2020, the Your COVID Recovery website was launched by the National Health Service of England with the aim of creating a practical tool that provides advice and support to individuals recovering from COVID-19. The website includes information on many of the key COVID-19 symptoms. To date, public use of the Your COVID Recovery website and user behavior remain unknown. However, this information is likely to afford insight into the impact of the website and most commonly experienced COVID-19 symptoms.

**Objective:**

This study aimed to evaluate public use of the Your COVID Recovery website, a digital health platform that provides support to individuals recovering from COVID-19, and determine user behavior during its first year of operation.

**Methods:**

Google Analytics software that was integrated into the Your COVID Recovery website was used to assess website use and user behavior between July 31, 2020, and July 31, 2021. Variables that were tracked included the number of users, user country of residence, traffic source, number of page views, number of session views, and mean session duration. User data were compared to COVID-19 case data downloaded from the UK government’s website.

**Results:**

During the study period, 2,062,394 users accessed the Your COVID Recovery website. The majority of users were located in the United Kingdom (1,265,061/2,062,394, 61.30%) and accessed the website via a search engine (1,443,057/2,062,394, 69.97%). The number of daily website users (n=15,298) peaked on January 18, 2021, during the second wave of COVID-19 in the United Kingdom. The most frequently visited pages after the home page were for the following COVID-19 symptoms: *Cough* (n=550,190, 12.17%), *Fatigue* (n=432,421, 9.56%), *Musculoskeletal pain* (n=406,859, 9.00%), *Taste and smell* (n=270,599, 5.98%), and *Breathlessness* (n=203,136, 4.49%). The average session duration was 1 minute 13 seconds.

**Conclusions:**

A large cohort of individuals actively sought help with their COVID-19 recovery from the website, championing the potential of this tool to target an unmet health care need. User behavior demonstrated that individuals were primarily seeking advice on how to relieve and manage COVID-19 symptoms, especially symptoms of cough, fatigue, and musculoskeletal pain. COVID-19 rehabilitation programs should use the results of this study to ensure that the program content meets the needs of the post–COVID-19 population.

## Introduction

The COVID-19 pandemic, caused by SARS-CoV-2, created a public health emergency of international concern [1]. At the time of this writing, there have been over 22 million confirmed cases, 945,000 hospital admissions, and 175,000 deaths related to COVID-19 in the United Kingdom [2]. Globally, these figures are compounded, with more than 6.4 million deaths worldwide [3].

Symptoms of COVID-19 have been reported in the acute phase of the infection, short-term follow-up, and in the succeeding months [4-9]. The symptoms experienced at these time points have been catalogued among both individuals who were hospitalized due to COVID-19 and those who managed their infection in the community [6-9]. Individuals experiencing COVID-19 symptoms for more than 12 weeks post infection are considered to have post–COVID-19 syndrome or post–COVID-19 condition [10,11].

Commonly reported symptoms of COVID-19, irrespective of how the initial infection was managed, include fatigue, dyspnea, fever, headaches, loss of taste and smell, and musculoskeletal pain [4-9]. Together with the cognitive and psychological symptoms of COVID-19, these physical symptoms impact quality of life, health, and activities of daily living [6-8,12]. The World Health Organization, which proposed the definition of post–COVID-19 condition, acknowledge that these symptoms can fluctuate in terms of number and severity [11].

Recovery and ongoing symptom burden from COVID-19 has been reported in a number of large cohorts [6-9]. These studies describe blunted recovery for a large proportion of individuals. Indeed, symptoms persist for months in up to 92% and 36% of hospitalized and nonhospitalized individuals, respectively [6-9]. The Post-Hospitalisation COVID-19 (PHOSP-COVID) study, a large multicenter follow-up study in the United Kingdom, reported that ~6 months post hospital discharge for COVID-19, only 29% of individuals felt fully recovered [6]. Several characteristics are associated with an increased likelihood of post–COVID-19 condition, including increasing age and BMI and female sex [13]. Thus, there is a pressing need to provide reliable and accurate information to support recovery from COVID-19 at scale.

Digital health platforms, including websites and smartphone apps, represent an opportunity to provide rapid access to education and self-management tools to millions of people, overcoming the constraints of health care professional time and social distancing rules. Due to these characteristics, the pandemic has witnessed a rapid increase in the application of digital technologies that aim to mitigate the impact of COVID-19 on individuals and health systems [14]. During the early stages of the pandemic, digital technologies were implemented to prevent and detect COVID-19 infections; for example, Singapore and Taiwan introduced a mobile contact tracing app to reduce the transmission of SARS-CoV-2 [15,16]. Digital tools were also used to host digital consultations and monitor the vital signs and symptoms of patients managing their COVID-19 infection in the community [17,18]. However, no digital tool was available in the United Kingdom, which provided general information on all elements of recovering from COVID-19, including the physical, emotional, and psychological well-being.

Therefore, in reaction to the urgent need to support individuals throughout their COVID-19 recovery, the Your COVID Recovery website was launched on July 31, 2020 [19]. Created by a research team at University Hospitals of Leicester in collaboration with health care professionals, professional bodies, patients with COVID-19, and the National Health Service (NHS) of England, this website is freely accessible to everyone with internet access. The website contains information on the effects of COVID-19 on the mind and body, including guidance on how to relieve and manage >15 COVID-19 symptoms. Information on how to manage daily activities, cope with grief and bereavement, return to work, and access additional support is also available on the website.

The use of this digital health platform is yet to be assessed but may inform whether it effectively targets an unmet health care need. Understanding the health issues in individuals recovering from COVID-19 is paramount to developing and implementing effective treatment strategies. Characterizing the knowledge seeking behaviors of individuals recovering from COVID-19 is likely to provide insight into the symptoms individuals are experiencing and the health care support required. Currently, information seeking behaviors in relation to COVID-19 remain largely unknown [20]. Therefore, the aim of this study was to assess usage patterns of the Your COVID Recovery website to understand public use and user behavior during its first year of operation.

## Methods

Aggregate data sourced from Google Analytics were used to assess website use and user behavior. Google collects and links actions via a JavaScript code installed on the website. All extracted data were anonymous. The “Privacy and Cookies” page on the Your COVID Recovery website outlines how information on user activity is collected and how visitors can opt out of data collection.

The following data were collected to assess website use and user engagement: number of users, user country of residence, traffic source, number of page views, number of sessions per user, and mean session duration. A page view occurs when a single page is loaded or reloaded in a browser, whereas a session refers to a group of interactions with the website, which take place within a set time frame (sessions end after 30 minutes of inactivity). User data were compared to COVID-19 case data downloaded from the UK government’s website [2].

## Results

### Website Users

In one year (between July 31, 2020, and July 31, 2021), the Your COVID Recovery website had 2,062,394 users from 187 countries. More than half of these users (1,265,061/2,062,394, 61.30%) were from the United Kingdom, with 14.27% (294,321/2,062,394) of all users located in London (Table 1).

Users were primarily acquired from an organic search (1,443,057/2,062,394, 69.97%; traffic from search engines), a direct search (290,591/2,062,394, 14.09%; entering the website address in a browser search bar), through referrals (269,142/2,062,394, 13.05%; traffic from other websites providing a website link), and social media (58,160/2,062,394, 2.82%). There were no paid advertisements for the website and 1444 of 2,062,394 (0.07%) users accessed the website via email. The top 3 referral websites were owned by the NHS, British Broadcasting Corporation, and British Heart Foundation. Almost all users used a mobile phone (1,559,072/2,062,394, 75.59%) or desktop computer (437,190/2,062,394, 21.20%) to access the website.

**Figure 1:**
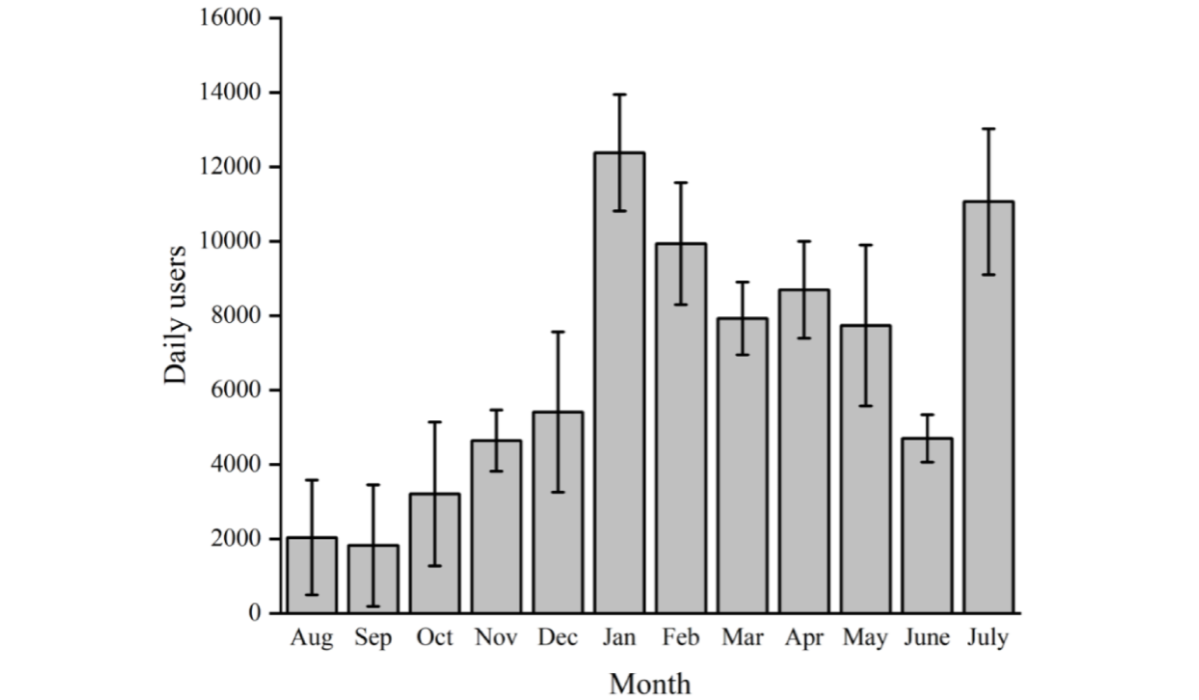
The number of daily users who accessed the Your COVID Recovery website each month. Data presented as mean (SD) values.

**Figure 2:**
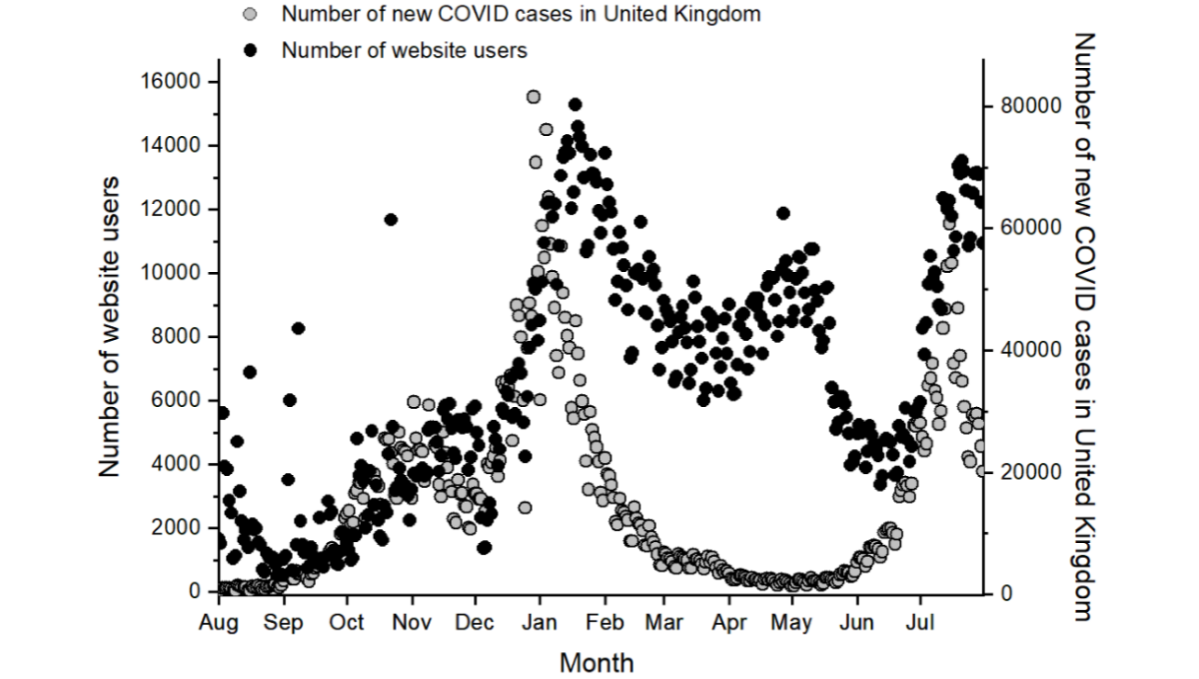
Number of daily website users and new COVID-19 cases in the United Kingdom between July 31, 2020, and July 31, 2021. COVID-19 case data are obtained from the UK government website for data and insights on COVID-19 [2].

The mean number of daily users who accessed the website each month is shown in Figure 1; the number of users (n=15,298) peaked on January 18, 2021, during the second wave of COVID-19 in the United Kingdom (Figure 2).

**Table 1:** Countries form which the users accessed the Your COVID Recovery website.

Country	Users, n (%)
United Kingdom	1,265,061 (61.30)
United States	334,162 (16.20)
India	203,588 (9.87)
South Africa	30,987 (1.50)
Canada	23,431 (1.14)
Ireland	11,789 (0.57)
Philippines	11,303 (0.55)
Pakistan	11,283 (0.55)
United Arab Emirates	10,954 (0.53)
Other countries^a^	156,309 (7.58)

^a^Countries that account for less than 0.5% of total users. Data were not available for 0.17% of users.

### User Behavior

**Figure 3:**
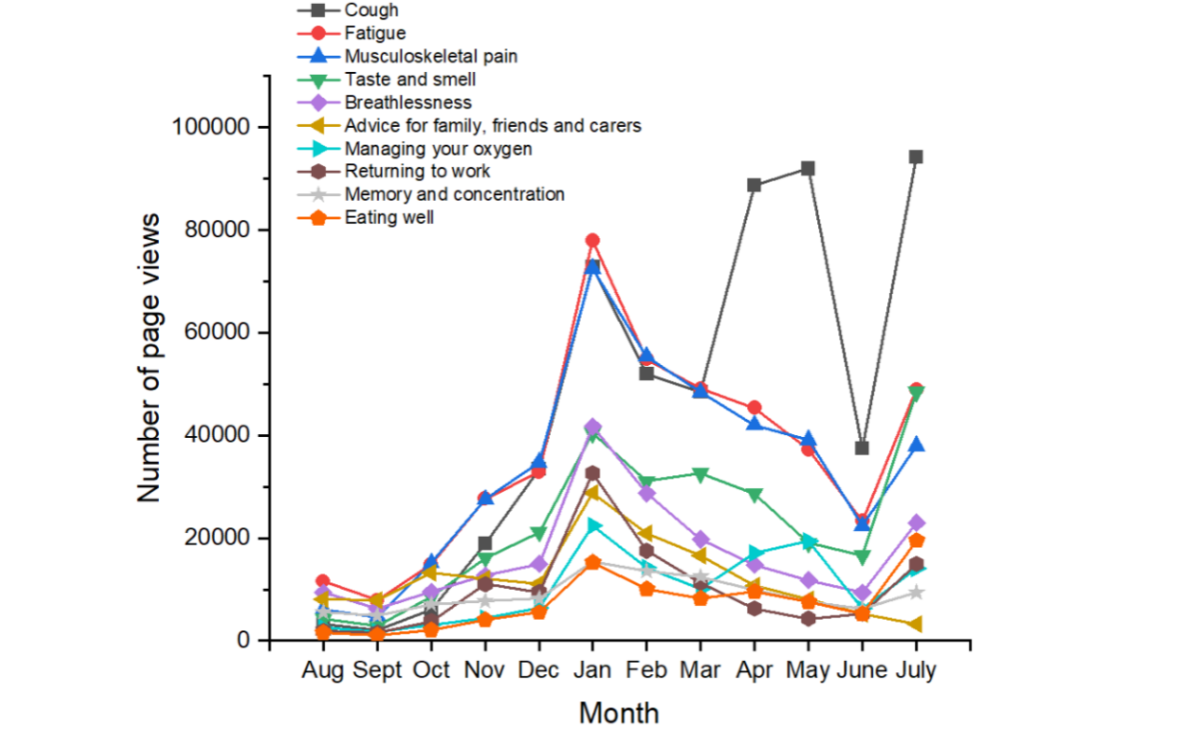
Number of page views received each month by the 10 most viewed pages.

The Your COVID Recovery home page had the highest number of page views (1,300,622/4,522,182, 28.76%). This page contains information on the purpose of the website and links to all other pages. After the home page, the following pages received the most views: *Cough* (550,190/4,522,182, 12.17%), *Fatigue* (432,421/4,522,182, 9.56%), *Musculoskeletal pain* (406,859/4,522,182, 9.00%), *Taste and smell* (270,599/4,522,182, 5.98%), and *Breathlessness* (203,136/4,522,182, 4.49%). All these pages provide advice on how to relieve and manage a specific COVID-19 symptom and when to seek further help. Shifts in user behavior occurred throughout the study period; Figure 3 shows monthly page views for the 10 most viewed pages.

### User Engagement

During the first year of operation, there were 2,642,377 sessions. Most users (2,068,638/2,642,377, 78.29%) visited the website once (Table 2). On average, users viewed 1.71 pages per session with an average session duration of 1 minute 13 seconds (Table 3).

**Table 2:** Frequency of sessions.

Session frequency	Sessions, n (%)
1	2,068,638 (78.29)
2	331,462 (12.54)
3	98,563 (3.73)
4	43,872 (1.66)
5	24,128 (0.91)
6	15,185 (0.57)
7	10,402 (0.39)
8	7441 (0.28)
9-14	20,862 (0.79)
15-25	10,955 (0.41)
26-50	6002 (0.23)
51-100	2644 (0.10)
101-200	1367 (0.05)
≥201	856 (0.03)

**Table 3:** Duration of sessions.

Session duration (minutes)	Sessions, n (%)
≤1	2,204,368 (83.42)
>1 to ≤3	175,896 (6.66)
>3 to ≤10	167,892 (6.35)
>10	94,221 (3.57)

## Discussion

### Principal Findings

This study was the first to investigate public use of the Your COVID Recovery website and user behavior during its first year of operation. The website contains a myriad of resources, including information on how to manage daily activities, get moving again, care for friends and family, cope with grief and bereavement, and relieve and manage >15 COVID-19 symptoms. Our data show that more than 2 million individuals accessed the website in a 12-month period, primarily to view information on COVID-19 symptoms. Taken together, these findings highlight the potential of this novel tool to help millions of people with their COVID-19 recovery without placing additional strain on health care services.

### Number of Website Users

As previously stated, over 2 million users from 187 countries accessed the Your COVID Recovery website in the study period. Our results show that a large cohort of individuals were actively seeking support with their COVID-19 recovery and were willing to receive this support via a digital health platform. In line with this behavior, other studies have reported an increase in the use of digital social platforms to look up information during the pandemic, potentially due to the isolation imposed by the pandemic [21,22]. The authors of this paper are unaware of any similar health care platforms, negating comparisons between user numbers. However, the large number of website users in this study aligns with the public’s high level of willingness to use other digital tools generated during the pandemic, such as contact tracing apps [23]. We also report that the majority of users accessed the website via organic search (ie, through a search engine)—an observation that is consistent with the information seeking behaviors of university students in Germany during the early stages of the COVID-19 pandemic [24].

Generally, changes in the number of daily website visitors mirrored those in the number of UK COVID-19 cases (Figure 2). However, several spikes in the number of daily website visitors occurred between August 2020 and November 2020 (Figure 2). Throughout the study period, the website was shared by collaborators, charities, media outlets, and members of the public on an ad hoc basis via news reports, email, and social media platforms; there was no marketing plan in place. The acquisition report on Google Analytics shows that the majority of the aforementioned spikes in website visitors were consequent to an increase in referrals from media outlets. A spike in website users also occurred during April 2021, approximately 10 weeks after the peak of the second wave of COVID-19 in the United Kingdom. At this time, the number of new daily COVID-19 cases was less than 1000 (Figure 2). This suggests that the UK public accessed the website in response to acute COVID-19 infections and persistent COVID-19 symptoms.

### User Behavior

Users were primarily seeking information on how to relieve and manage COVID-19 symptoms. The top 5 pages viewed suggest that individuals would like more information on the following symptoms: *Cough*, *Fatigue*, *Musculoskeletal pain*, *Loss of taste and smell*, and *Breathlessness*. Consistent with these page views, 5 months after a COVID-19–related hospitalization, 92.8% (632/855) of patients had a persistent symptom, and the most common symptoms were muscle pain, fatigue, and physical slowing down [6]. Likewise, in a cohort of hospitalized and nonhospitalized patients, 4 months after a COVID-19 infection, the most common symptoms were fatigue, breathlessness, and muscle ache [25]. Thus, page views on the Your COVID Recovery website align with the most frequently reported COVID-19 symptoms.

The number of views received by the top 10 pages each month tended to follow the same trend, except for cough (Figure 3). The factors underpinning the increase in cough page views between March and May 2021 are unclear. The increase in cough page views does not coincide with an increase in page referrals from a single source, suggesting that it is not the result of an external organization promoting the web page. Furthermore, the authors are unaware of any events, announcements, or government initiatives within this period, which may have propagated page views. Thus, it is tempting to speculate that the observation may reflect an increase in individuals experiencing a COVID-19–related cough between March and May 2021. However, further work is needed to investigate this notion and resolve the cause.

Rehabilitation services should ensure that information on the most viewed symptoms is included in their programs to meet the demands of the post–COVID-19 population. Health care professionals involved in acute COVID-19 care and the post–COVID-19 condition care pathway should also discuss the most commonly sought symptoms with patients and signpost patients to the website for symptom support.

### Limitations

The limitations of this study relate to the use of Google Analytics. A new client ID is created each time a user deletes the browser cookies, uses a different browser, or switches devices. Subsequently, the same user can be counted as a new user [26]. Nevertheless, this parameter provides a strong indication of website use.

User demographics can only be collected from users who are logged into their Google account when visiting the website. In this study, demographics were collected for 366,281/2,062,394 (17.76%) of users and were, therefore, not reported. This information is crucial to understand the true use of the website and develop initiatives that target poorly engaged populations. Previously, several factors have been linked to information seeking about COVID-19, including living in an urban area, perceived disease susceptibility, perceived disease severity, higher self-efficacy, and higher health literacy [20].

Pages on chest pain and headaches post–COVID-19 infection were added to the website on June 1, 2021, and included in the analysis. The popularity of these pages remains unclear due to the content only being available for 4 weeks of the study period. Finally, the website may have obviated the need for phone-based or physical appointments between health care professionals and individuals recovering from COVID-19, bridging the gap between demand and limited health care resources. However, to confirm this, further investigation is necessary.

### Conclusions

This study describes the usage patterns of the Your COVID Recovery website during its first year of operation. Over 2 million users accessed the website, demonstrating that individuals are keen to receive digital health care support with their COVID-19 recovery. Users primarily visited the website to attain information on how to relieve and manage COVID-19 symptoms, particularly cough, fatigue, and musculoskeletal pain.

## Abbreviations

NHS: National Health Service
PHOSP-COVID: Post-Hospitalisation COVID-19
